# Analysis of optimal solutions in article on linear programming

**DOI:** 10.1590/S1679-45082018CE4718

**Published:** 2018-09-10

**Authors:** José Sérgio Domingues

**Affiliations:** Departamento de Matemática, Instituto Federal de Minas Gerais, Formiga, MG, Brazil

Dear Editor,

Although published in 2003, the article “Linear programming applied to healthcare problems”^(^ ^1^ ^)^ is very up-to-date and important for the first contact with linear programming, particularly in the field of health. Congratulations to the author for this initiative. I analyzed this work to use it in a undergraduate degree in Mathematics. However, I observed the solutions obtained for the two problems described present inconsistencies that may lead readers with little experience to make mistakes. For the diet problem, the solutions found were *X*_1_ = 1.4 and *X*_2_ = 0.2. In reality, these values are approximations, since the real solutions are recurring decimals: *X*_1_ = 339/248 ≅ 1.37 and *X*_2_ = 41/248 ≅ 0.17. The values presented by the author show rounding to one decimal place, but this fact was omitted. Moreover, the value of the objective function for the optimal solution is described as *Z* = R$ 2.55, but it occurs only when the values used are the real ones. For the approximation, it would be *Z* = R$ 2.70, nearly 5.9% greater than the correct value. To give an idea of the effect the approximation causes, if *X*_2_ = 41/248 were used, the salad portion would be 82.66g, and not 100g as shown (approximately 21% greater than the correct value).

In the problem of resource allocation, the result indicates that the interventions 1, 3 and 5 should not be used. This means that *X*_1_ = *X*_3_ = *X*_5_ = 0, but the solution presented is: *X*_1_ = 1; *X*_2_ = 0.5; *X*_3_ = 0; *X*_4_ = 1; *X*_5_ = 0. Therefore, the value attributed to the variable *X*_1_ is wrong. In addition, the solution does not serve for the second problem restriction, which should be ≤40 and is equal to 80. In the Discussion section, the author correctly presents the solution, but undoubtedly, it is confusing for beginners studying linear programming.
